# Development of an mHealth Application for Women Newly Diagnosed with Osteoporosis without Preceding Fractures: A Participatory Design Approach

**DOI:** 10.3390/ijerph15020330

**Published:** 2018-02-13

**Authors:** Pernille Ravn Jakobsen, Anne Pernille Hermann, Jens Søndergaard, Uffe Kock Wiil, Jane Clemensen

**Affiliations:** 1 Center for Innovative Medical Technology, Odense University Hospital, 5000 Odense C, Denmark; ukwiil@mmmi.sdu.dk (U.K.W.); jane.clemensen@rsyd.dk (J.C.); 2 Institute of Clinical Research, Faculty of Health Sciences, University of Southern Denmark, 5000 Odense C, Denmark; 3 Department of Endocrinology, Odense University Hospital, 5000 Odense C, Denmark; pernille.hermann@rsyd.dk; 4 Department of Public Health, Research Unit of General Practice, University of Southern Denmark, 5000 Odense C, Denmark; jsoendergaard@health.sdu.dk; 5 SDU Health Informatics and Technology, The Maersk Mc-Kinney Moller Institute, University of Southern Denmark, 5230 Odense M, Denmark; 6 HC Andersen Children’s Hospital, Odense University Hospital, 5000 Odense C, Denmark

**Keywords:** mHealth, application, app, participatory design, osteoporosis, women

## Abstract

mHealth is a useful tool to improve health outcome within chronic disease management. However, mHealth is not implemented in the field of postmenopausal osteoporosis even though it is a major worldwide health challenge. Therefore, this study aims to design and develop an mHealth app to support women in self-management of osteoporosis when they are diagnosed without preceding fractures. Participatory design is conducted in three phases. Based on identified needs in the first phase, a prototype is designed and developed in an iterative process in the second phase before the mHealth app is tested in the third phase. This paper focuses on the user activities in phase two and describes how a team of researchers, women, physicians, healthcare professionals, and app designers are involved in the participatory design process. The study shows that participatory design is a viable approach when developing an mHealth app for women with asymptomatic osteoporosis. Results obtained from the workshops and laboratory tests demonstrate the importance of feedback from users in the iterative process, as well as the participation of users and app designers in workshops and laboratory tests to enable mutual learning when developing new mHealth solutions. The regular member-checks and involvement of users helped to identify challenges associated with providing healthcare services through an app.

## 1. Introduction

Mobile health (mHealth) is a rapidly growing area of healthcare delivery, in which mobile devices, particularly via smartphone applications (apps), are used to support medical and public health practice [1]. Studies have shown that mHealth apps may be useful tools for patient self-management, as well as for facilitating improved communication between patients and health care providers [2]. Furthermore, studies have shown that within chronic disease management, the use of apps in mHealth has the potential to improve health outcomes [3]. However, mHealth has not yet been deployed in the field of osteoporosis even though the disease has been recognized as a worldwide challenge [4]. Osteoporosis (low bone density) is a common chronic bone disease associated with increased risk of fractures and hence reduced quality of life and fracture-related morbidity and mortality. The disease poses an increasing economic burden for healthcare systems all over the world [5,6]. Osteoporosis is estimated to afflict more than 41% of women and 18% of men aged 50 years and older of whom 1 in 3 is predicted to have a fracture [5,7].

It is possible to diagnose osteoporosis prior to fracture by measuring the bone mineral density (BMD) by performing a Dual-energy X-ray Absorptiometry (DXA) scan [8]. Effective prevention is available in the form of medication, calcium supplements, dietary changes, and weight-bearing exercise [9]. This is highly relevant for postmenopausal women at risk of osteoporotic fractures to initiate secondary prevention [5]. However, studies show that women diagnosed with asymptomatic osteoporosis have difficulties in perceiving and interpreting the diagnosis, their current risk of fractures, and in managing everyday life with the disease [10,11,12,13]. A newly published study concluded that there is a need for improved support for these women to help them in self-management of their disease [14]. In addition, studies have shown that a higher degree of compliance to treatment recommendations can significantly reduce the number of fractures [15,16].

mHealth can support chronic disease self-management and provide personalized, localized, and on-demand interventions [1,17,18]. Peeters, Wiegers, and Friele (2016) found in their scoping review that patients that use technology have a better understanding and more knowledge of their disease. Most of the included studies in the review show effects of technology on the level of increased patient competence [19]. Additionally, a number of studies have underlined that a well-informed patient is more likely to participate in healthy behaviours and to better manage their conditions. Hence, an mHealth solution may adequately meet the challenges faced in relation to osteoporosis [20]. Therefore, this study aims to design, develop, and test an mHealth app for women newly diagnosed with osteoporosis without preceding fractures to support the women in treatment decision-making and their self-management of the disease. Involvement of users is of high importance when developing new technologies to be implemented in healthcare. One method to engage users in the design and development process is to use participatory design [21]. Thus, participatory design is increasingly being used in the development of new technological solutions in different areas of healthcare with promising results [22,23,24,25,26].

This paper describes the user activities in the participatory design process, in which we have engaged a team of researchers, women, physicians, healthcare professionals, and app designers in the design and development phase.

## 2. Materials and Methods

**Figure 1:**
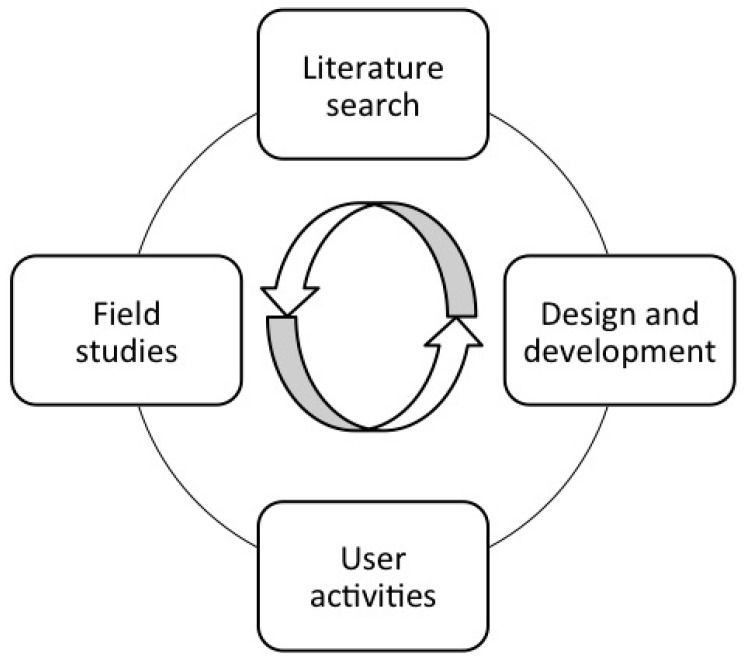
The ongoing and parallel activities in the participatory design process. Inspired by Clemensen [28].

Participatory design has its roots in action research and combines the use of various methods and activities such as field studies, literature search, user activities, design, and development [23]. The different methods and activities are employed to reflect the aim of the study. Those ongoing and parallel activities during the research process are shown in Figure 1. The user activities and the design and development activities have a more iterative nature than field studies and literature search. It is not possible to predetermine the number of iterations, which elements in the process that are iterated and what the various activities exactly should consist of. That depends on the process, the technological solution, and the necessary organizational changes in each study [27].

In a participatory design study involvement of users and health IT designers is essential [27]. When they collaborate, mutual learning is an ongoing activity. Designers need knowledge of the users’ needs and behaviour. Users need knowledge of potential technological options, as well as of how these options can be provided. Designers are the source of this knowledge, as well as of relevant design expertise [21,29]. The use of participatory design in health research is an iterative process usually performed in phases, which involves identification of users’ needs, design and development of prototypes, testing, retesting, and evaluation [21,27,30]. Technologies developed through participatory design projects are often more successfully implemented, since the end-users are involved in the process from identification of needs to design and development of solutions [31]. An example of this is a project using participatory design involving patients with diabetic foot ulcers, which resulted in home treatment of ulcers through a telemedicine solution that is now being implemented nationally in Denmark [32].

This study is approved by the Danish Data Protection Agency and approved by hospital management (J.no. 2008-58-0035). Due to the design and the non-biomedical character of the study, it was not necessary to get approval from the local ethical committee. The participants in the team received verbal and written information about the project and their right to withdraw before signing the informed consent.

### 2.1. Research Design

**Figure 2:**
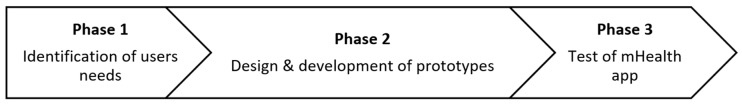
The participatory design process in the study.

We conducted the study in three phases: (1) Identification of needs among users from March 2015–September 2015; (2) design and development of prototypes from September 2015–August 2017; and finally (3) a test initiated in August 2017 and finished in February 2018. The process is illustrated in Figure 2.

In the first phase a literature search, field studies (10 h), semi-structured interviews with women (*n* = 17) and physicians (*n* = 6), and one focus group with women (*n* = 3) were performed to identify needs among users. This paper describes phase 2, which consists of user activities such as generation of ideas, design of mock-ups, development and test of prototypes throughout three workshops (*n* = 6 h), and two laboratory tests (*n* = 4 h). Furthermore, the phase consisted of continuous evaluation of the design through semi-structured interviews and field studies to maintain relevance to end-users.

### 2.2. Setting and Participants

We initiated a team in November 2015 as recommended when conducting a participatory design study [31]. The team consisted of researchers (*n* = 2), women newly diagnosed with osteoporosis (*n* = 6), physicians (*n* = 4), laboratory specialists *(n* = 2), a nurse (*n* = 1), a dietician (*n* = 1), and app designers (*n* = 2). The aim was to establish a team representing patients and stakeholders from all levels within the field of early detection of osteoporosis in women. All participants in the second phase have participated in the first phase of the study and were selected based on their contributions in the first phase. The women and the healthcare professionals were recruited from the Department of Endocrinology at a Danish University Hospital and the general practitioners (GPs) from nearby medical practices. Their roles, characteristics, and attendance in workshops and laboratory tests are outlined in Table 1.

### 2.3. The Participatory Design and Development Process in Phase 2

The user activities in phase 2 were based on the identified needs in phase 1. Briefly, women need targeted and tailored information about osteoporosis with focus on the benefits of detecting the disease before fractures have occurred. Furthermore, they want to be better prepared for the GP visit to be able to participate in treatment decision-making. Finally, they ask for information about how to handle daily life with osteoporosis with focus on how to strengthen the bones through diet, exercise, and calcium and vitamin D supplements. The women agreed that medical treatment should not be served as the only way to manage the disease [33]. The physicians think that if the women could be better prepared for the GP visit, the treatment decision-making would be more like a dialogue based on the women’s preferences. Hence the women would be able to better manage the disease in their daily life without assuming a sickness role.

With those needs in mind, we designed and developed an mHealth downloadable app called “My Osteoporosis Journey” in an iterative participatory design process. The participatory design process was organized in cycles with an iterative and agile software development process, as recommended by Simonsen and Robertson [31]. We used creative processes in workshops, such as “5 Whys” and “Fish Bowl” games. Those games are useful when there is a need for a deeper understanding of the problem and its context so that the participants can get the greatest leverage out of solving it [34]. Throughout the design and development phase, we focused on continuous feedback from users. We collected data through field studies and semi-structured interviews. Workshops and laboratory tests were video recorded and pictures were taken. Field notes were taken continuously.

## 3. Results

**Figure 3:**
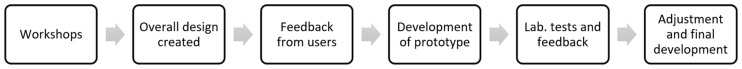
The user activities and the design and development activities in phase 2.

Phase 2 of the participatory design process was initiated with three workshops with idea generation and design activities. The workshops were followed by a period of development with continuous user activities and development before laboratory tests at the hospital were conducted. Finally, a prototype was fully developed and prepared for the test in phase 3. The participatory design process in phase 2 is illustrated in Figure 3.

The aim of the mHealth app is to support the women in engaging in treatment decision-making and self-management of the disease. In the following, we describe the way from needs to the solution with focus on the user activities and the iterations throughout the design and development process.

### 3.1. Workshops

Three workshops were held at a Danish Health Innovation Centre. The workshops were led by three experienced innovation consultants to achieve a comprehensive understanding of the needs and demands of the solution. Every workshop lasted two hours.

#### 3.1.1. Workshop 1

**Figure 4:**
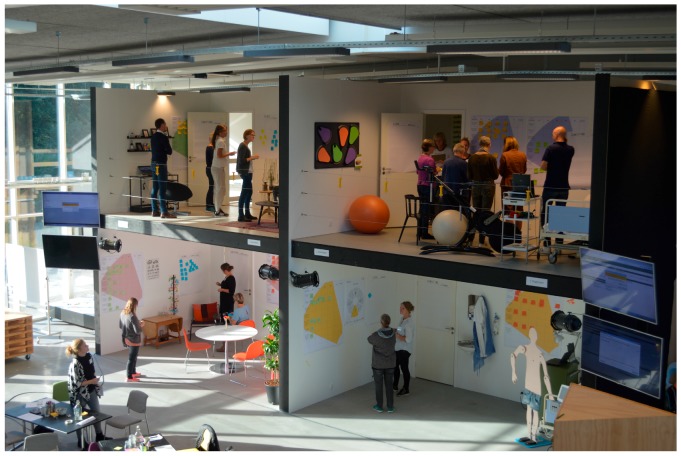
This picture is from the first workshop in a Plug and Play Lab at a Danish Health Innovation Centre. The participants generated ideas and concepts based on the identified needs in the four rooms that represented different situations in the osteoporosis pathway.

The workshop was held in September 2015 in a Plug & Play Lab at the Danish Health Innovation Centre. The aim of the first workshop was to generate ideas based on the identified needs in phase 1. 16 members of the team participated (Table 1). Four rooms were created, representing (1) the scanning situation at the hospital, (2) the time when waiting for the result, (3) the GP visit, and (4) the daily life with osteoporosis (Figure 4). The participants were divided into four groups with a mix of patients and healthcare professionals in each group. Firstly, the identified needs were presented, and afterwards, 5 Whys was used as a tool for seeing the bigger picture of the needs of the users. Finally, the groups generated ideas and concepts. The app designer was observing and listened to the ideas and concepts being generated.

##### Findings

All four groups had ideas regarding how to support women in being prepared for the treatment decision-making. They generated ideas about an app that could give the women the result of the DXA scan in a more effective, fast, and understandable way. Ideas were also generated regarding easily understandable information about osteoporosis and how it should be given and when. The ideas concerned videos, pictures, texts, and case stories with a focus on the positive benefits of early detection of osteoporosis. The women emphasized that less attention should be paid to chronic disease management and medication as the only treatment option. They highlighted that they did not want to be placed in a sickness role. The healthcare professionals supported this approach, since they agreed that osteoporosis was not a serious disease when no fractures had occurred. That could be videos about bone-healthy lifestyle and how to increase intake of vitamin D and calcium. Finally, ideas were generated about a digital platform with information about osteoporosis with a health promotion perspective. The workshop was video recorded.

After the workshop, the app designers started developing wireframes of an mHealth app. The content was based on the findings from the workshop. Drafts of wireframes were presented to the researchers, and feedback was given before wireframes were fully designed and presented in the next workshops.

#### 3.1.2. Workshop 2

The workshop was held in November 2015 after the first draft of wireframes was finished. 8 members of the team participated (Table 1). The aim of the second workshop was to present the wireframes for the healthcare professionals and discuss further design and development of an mHealth app with a focus on how and where to implement it. The participants were divided into two groups with the healthcare professionals in one group and the researchers and app designer in another group. Firstly, the wireframes were presented to the participants. Fishbowl was used as a tool to discuss different perspectives based on the first workshop and the wireframes developed. The two groups shifted from being a discussion group to an observer group. When a group was a discussion group, the observer group was not allowed to interrupt the discussions going on. The content of the wireframes was discussed with a focus on further designing of the mHealth app. Field notes were taken and the workshop was video recorded.

##### Findings

The physicians supported the idea of developing an app that could provide the women with the result of the DXA scan before the GP visit. The physicians discussed whether it should be the GP or the hospital introducing the app for the women. This was combined with a discussion in the group about where the app should be implemented. They agreed that the app should be introduced by the Department of Endocrinology at the University Hospital, with this same department performing the scan. The laboratory specialist suggested that it could be introduced in connection with the DXA scan at the hospital. The GP suggested that the app could be used as a way of dividing the GP visit into two parts. The first part of the GP visit should consist of giving the woman her result of the DXA scan and treatment recommendations. Then, the woman could prepare at home before the second GP visit, in which a treatment decision-making process should take place. The first idea of how to provide the woman with the result of the scan through an app emerged.

After the workshop, the app designers adjusted the wireframes. It was decided to design a general knowledge base in the app, with targeted and tailored information about osteoporosis in women without preceding fractures. This should be freely available in App store and Google play without login. Furthermore, the app should contain an individual part with secured login, in which information regarding the result of the DXA scan should be presented.

#### 3.1.3. Workshop 3

**Figure 5:**
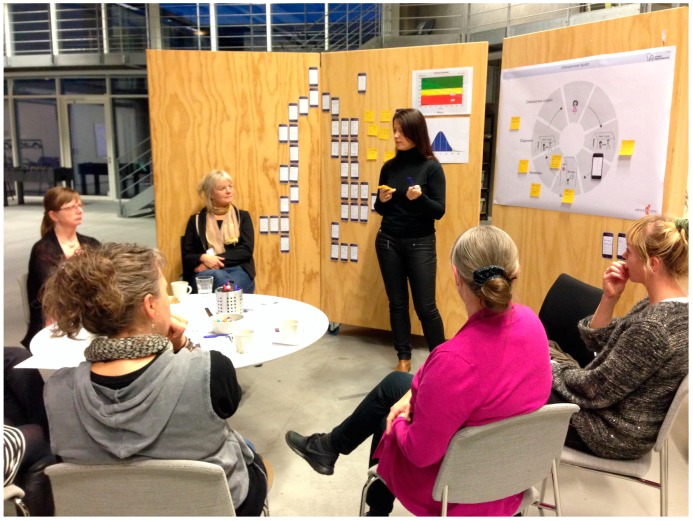
This picture is from the third workshop. Women and researchers from the team discussed how the result of the DXA scan should be given through the app. The substance in the general knowledge part and in the individual part of the app was discussed. Ideas were generated and wireframes were further designed.

This workshop included 5 women from the team and the two researchers (Table 1). It was held 14 days later than workshop 2. The aim of the third workshop was to discuss the overall design and content of the app presented through the wireframes. Furthermore, it was important to discuss how and when the result of the DXA scan should be given to the women through the app and how and when the app should be presented to the women. We worked in creative processes with the wireframes, and the women contributed expanding ideas about bone-healthy lifestyle and ways of handling osteoporosis in daily life through the app (Figure 5). Field notes and pictures were taken.

##### Findings

The women agreed that the result of the DXA scan should be presented as an easily understandable text message and supplemented with a video in which the result of the scan was explained on a graph with green, yellow, and red colours. However, it was important to explain the implications of the result of the scan and the risk of fractures in a way that will not place the women in a sickness role unnecessarily. They asked for a way of calculating the risk of fractures, since one woman has tried calculating her risk online and found that it was low, which will have had a calming influence on her interpretation of the diagnosis. They asked for an overview of their bone scans so that they easily could follow the progression of the disease every time they have had a DXA-scan. The knowledge base of osteoporosis should focus on osteoporosis as a common condition instead of a chronic bone disease. Furthermore, the focus should be on the benefits of detecting the disease before fractures have occurred. They suggested that some of the headlines in the app could be “how does it feel” instead of “symptoms” and “how do I strengthen my bones” instead of “treatment”.

After the workshops, the overall design of the app was sketched. The app designers described the functionality of the app by developing mock-ups on print in collaboration with the researchers. The mock-ups illustrated the overall content of the app.

### 3.2. The Design and Development Phase

The mock-ups were printed and were presented to 4 women in the team in February 2016. They were interviewed in their own home and feedback was collected regarding the overall design of the app before the development was initiated. Overall the women were positive towards the content and how it covered the identified needs from the workshops. Especially, they liked the opportunity of having the result of the scan as a text message through the app prior to the GP visit. They highlighted positively the general knowledge part of the app with targeted and tailored information about osteoporosis with a focus on the benefits of detecting the disease before fractures have occurred. The idea of introducing the app to the women before having the result of the scan was supported by the women, since they expressed that the need for information occurred as soon as they were referred to a DXA scan. The overall focus in the app to support women in self-management of osteoporosis regarding exercise, diet, and intake of calcium, vitamin D, and medication was positively received. The women emphasized that it was praiseworthy that medication was not served as the only solution, and that the app gave them the opportunity to either supplement or substitute medication with bone-healthy lifestyle.

Some new ideas were generated. The women asked for stories in the app, with women who have been diagnosed with osteoporosis without preceding fractures to get some inspiration of how they have handled it. Furthermore, the women asked for videos, with one of the hospital physicians explaining what osteoporosis is and how to interpret the result of the DXA scan. New ideas regarding reminders of exercise, intake of medicine, and next scanning dates were also generated.

The interviews were transcribed and sent to the app designers. Then, the development of the first prototype was launched in April 2016. The development process moved in iterative cycles in close collaboration between the researchers, the healthcare professionals at the Department of Endocrinology, and the app designers.

An mHealth app was developed based on a platform at the University Hospital called “My Patient Journey”. “My Patient Journey” is a platform developed to facilitate digital communication between the hospital and patients. It is a personal access point for patients involved in a number of specific treatment processes at the hospital, as well as for patients with chronic conditions. The platform consists of a backend and a web-based clinician interface and the new patient app called “My Osteoporosis Journey”. The app is a cross-platform app and is designed for both iPhone (iOS), Android, and Windows. Patients can also use a Web-based interface.

All the content in the app was prepared by the physicians at the department and specialized healthcare professionals within the field of osteoporosis. The researchers in the team led the process.

### 3.3. Laboratory Tests

The development process aimed at developing a prototype that should be tested at the hospital. Two laboratory tests were conducted.

#### 3.3.1. Laboratory Test 1 at the Hospital

**Figure 6:**
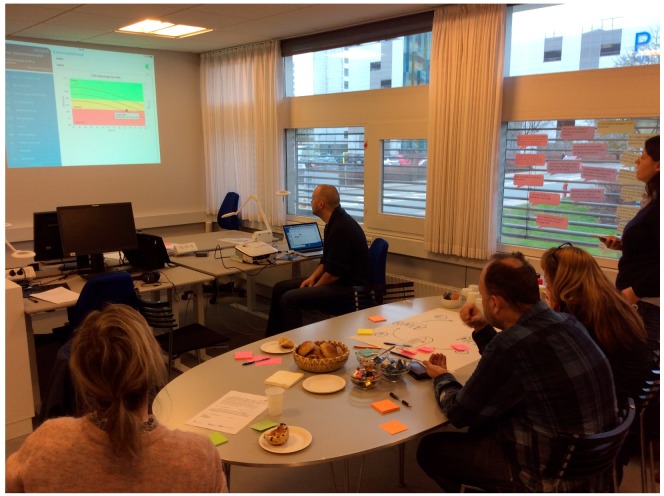
The first laboratory test at the hospital. We tested whether the result of the scan is send through the app, and how the result was illustrated in the graph. We also tested whether the planned material was released if a DXA scan showed that the osteoporosis was present.

In March 2017, the first prototype was finished and a laboratory test was held in a meeting room at the hospital. Six members of the team participated (Table 1). The aim was to test the app at the hospital and to find out whether the app functioned as planned. The process in which the app should be used to support the women in treatment decision-making and self-management of osteoporosis was reviewed. We tested whether the result of the scan was sent from the hospital through the app after the laboratory specialist had entered the T-score in the web-based clinician interphase. We also tested whether the planned material was released in the app if a DXA scan showed that the osteoporosis was present (Figure 6).

##### Findings

Every feature in the app functioned as planned. However, we found out that the woman would receive the result of the scan through the app immediately after the scan contrary to the GP. This shows that a scenario could arise where the woman could call her GP before the GP receives the description of the scanner and the treatment recommendations of the hospital and, therefore, not being prepared to see the woman. It became clear during the laboratory test that it was important to make sure that both the woman and the GP were prepared for the consultation and the treatment decision-making process. Hence, we decided to develop a functionality in the app that would automatically send a text message to the woman when the description of the scan was been sent from the hospital to the GP saying that now she could visit her GP to decide which treatment should be initiated.

An adjusted prototype was developed based on the findings of the laboratory test. This was tested in three women in the team. They were asked to download the app from app store and their previous DXA scan was send to them through the app. We asked them to go through the content of the app. We interviewed them in their homes after 1 week with a focus on usability and content. Furthermore, the app was presented for one of the GP’s in the team. The feedback was positive and there were no suggestions for adjustments. Based on their feedback, we decided that the app was fully developed and ready to test. Before the test, we decided to have one more laboratory test in the department at the hospital.

#### 3.3.2. Laboratory Test 2 at the Hospital

In May 2017, the prototype was tested at the Department of Endocrinology at the University Hospital. Seven members from the team participated (Table 1) together with the 4 employed laboratory specialists and 4 secretaries at the Department of Endocrinology. The aim was to test the app in a real-life setting, to make sure that everything functioned. Furthermore, the employees invited should get to know the app and be aware of their role in relation to the test in phase 3 (Figure 2). The laboratory specialists were introduced to the work procedure of how the result of the scan was entered in the platform and how the result was sent to the app.

##### Findings

The laboratory specialists emphasized that they did not feel qualified to answer questions that might occur after the women received the result of the scan. Since the result of the scan would be sent within 5 min, it was decided to develop a delay. This caused the scan result to be sent to the women the next morning.

### 3.4. The App “My Osteoporosis Journey”

**Figure 7:**
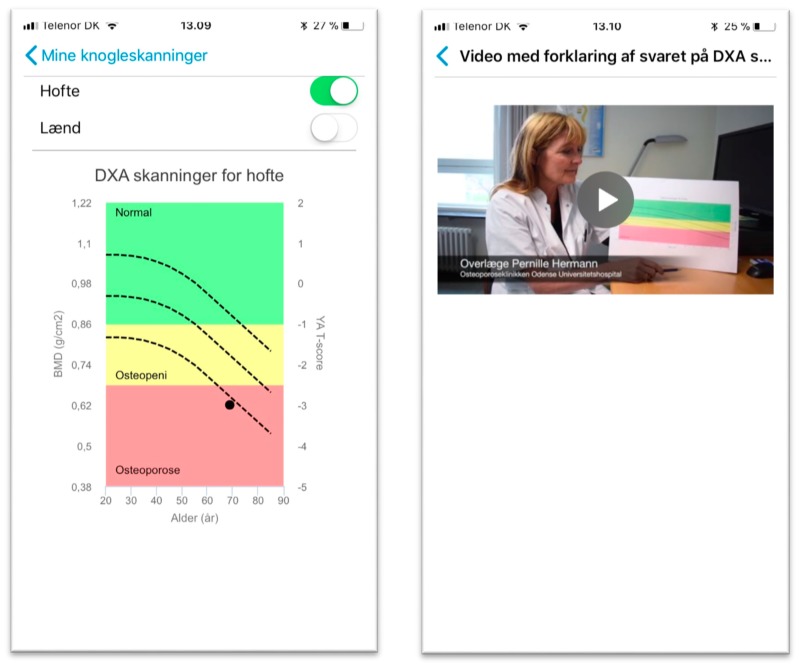
Screenshots from the menu item “My bonescans” (to the left) and the video with the chief physician explaining how to interpret the result of the DXA scan (to the right).

When a woman is admitted to the hospital, she is informed about the app and how to get it through App Store or Google Play Store in an information letter send from the Department of Endocrinology at the University Hospital. The app contains targeted and tailored information about osteoporosis and what the examination of the bones consists off and how it should be interpreted. The information is in both written and oral form using videos and pictures. The laboratory specialist offers to send the results of the scan through the app within 24 h after the woman is scanned. If the woman accepts, the laboratory specialists enter the result of the scan in the web-based clinician interphase and a message is automatically send with the result of the scan the next morning. The result of the scan is given both as a text message, a graph, and as a video in which the chief physician explains how to interpret the result of the DXA scan (Figure 7).

Open-ended questions aimed at helping the woman to prepare for the GP visit are available under the menu item “My preparation”. A message is automatically sent when the commentary of the scan formulated by the hospital physicians is sent to the GP to inform the woman that the GP is ready to see her. The woman can bring the app to the GP and note what they agreed upon regarding treatment and management of the disease. After being diagnosed, the woman can get support in self-management of the disease through information about bone-healthy diet and how to strengthen the bones with physical exercise in the menu item “My material”. The information is given through videos with exercise programs and a talk about bone-healthy diet. She can also calculate whether she is in need of calcium and vitamin D supplements. Finally, the woman is provided with reminders and notifications about medicine intake, daily self-management, and a reminder about when she should be scanned again to see how the disease progresses.

The next step is a test of the app at the Danish University Hospital (phase 3) to investigate whether mHealth apps could be a viable solution for women newly diagnosed with osteoporosis without preceding fractures. This will be the topic for future papers.

## 4. Discussion

In this study, we used participatory design to develop an mHealth app for women newly diagnosed with osteoporosis without preceding fractures. The aim was to develop an app that could support the women in treatment decision-making and self-management of the disease. By using participatory design, we were able to collaborate with women, healthcare professionals, and app designers in an iterative process. The first workshop revealed that the user requirements could be addressed by an app. Therefore, ideas were generated about contents of an app and how an app could be presented to the women in the following workshops. Every workshop was planned based on the findings of the previous workshop. We moved in iterations towards a solution that could meet most of the identified needs. The iterative process was of significant importance, since it gave the opportunity to revise the design until an acceptable solution was developed. This demonstrates the importance of using participatory design when designers and users collaborate in the design and development of new healthcare services. This is supported by the literature concerning the use of participatory design within health science [23,24,29]. Furthermore, a recent study concludes that integrating participatory design theory and methods can significantly improve interventions within healthcare [35]. The most ambitious part of the app “My Osteoporosis Journey” that was developed was the communication between the hospital and the woman providing the woman with the result of her DXA scan. This idea occurred during the creative workshops. To the best of our beliefs, this generation of ideas at an early stage in collaboration with the team was very important when it comes to transforming healthcare. The idea emerged when the women and the healthcare professionals collaborated using creative methods in the workshops. By using participatory design with emphasis on the generation of ideas and co-design with end-users, we were able to foster an mHealth app to support women in treatment decision-making and self-management of the disease. Continuous member-check among the users such as feedback through interviews and laboratory tests at the department helped us to develop a viable solution as recommended by Spinuzzi [36]. The last member-check before the second laboratory test convinced us that the prototype was finished and ready to test, since the feedback gained through interviews of three women and one of the GPs from the team was very positive. Then again, we decided to conduct the second laboratory test with the employees at the department where the app should be tested in the third phase. It turned out to be a good decision, since the feedback from the laboratory specialist resulted in an extra iteration, developing a delay of the answer of the scan. Likewise, an iteration based on member-check among the end-users was completed after the first laboratory test. The idea of sending a text message to the woman when the result of the scan was sent from the hospital to the GP was to make sure that both the woman and the GP were ready for the treatment decision-making. This was very important to both the GP and the women. If we have not conducted this iteration, we could have risked that the app developed would have met much resistance among GPs in the test phase. Likewise, Andersen et al. state that to succeed when developing mHealth prototypes, it is important to align or at least reconcile the concerns of patients with those of the clinicians and the healthcare systems. Otherwise, it is likely that either patients or clinicians will be reluctant to use the prototype [29].

When involving patients and clinicians in a participatory design process, it is important to be aware of the fact that they are engaged for different reasons and with different agendas. For the women in our study, the overarching goal of participating in the design and development phase was to become more familiar with their disease. For the healthcare professionals, in contrast, the primary goal was to be part of the transformation of healthcare services. However, it is important to be aware of the relevance of engaging all stakeholders in the field, and that users are not only patients but also healthcare professionals. This is supported by other studies in our research group using participatory design within health science [22,37,38].

Slomian et al. are some of the only ones requesting more research in the use of information and communication technology in the field of osteoporosis to handle the challenge in the healthcare system of ensuring adequate resources to support individuals with chronic diseases [20]. There have been many encouraging studies using mHealth apps in the field of most other chronic diseases. Since osteoporosis is an under prioritized disease, the transformation of healthcare has not started yet in this field. Thus, so far our study is the first study using participatory design to develop an mHealth app for women newly diagnosed with osteoporosis to support them in treatment decision-making and self-management of the disease. We are convinced that mHealth can be used to transform the way healthcare is delivered in the field of osteoporosis. When it comes to asymptomatic osteoporosis, which is most common in women, it is of high importance to both the women and the healthcare professionals not to place them in a sickness role unnecessarily. However, prevention of the first fracture is important, since the first fracture increases the risk of further fractures [39]. Therefore, the importance of supporting women in self-management of the disease in their daily life is overwhelmingly necessary. Encouraging and facilitating engagement and self-management among the women could decrease the risk of fractures and help them in handling their diagnosis without assuming a sickness role.

### Strengths and Limitations of Using Participatory Design

According to Kushniruk and Nøhr, the participatory design should ideally be initiated in the early phases of the design process [21]. In this study, we started the participatory design process by identifying needs in phase one before starting the design and development in phase two. This is one of the strengths of the study, since the identified needs were the underlying basis for the workshops, and the workshops were the underlying basis for the laboratory tests.

Since we were moving in iterations towards the final prototype with constant member-checks through active involvement of end-users at the department, the prototype is close to the stage of implementation. This is the result of the continuous involvement of the healthcare professionals at the department at the University Hospital who are initiating the change by introducing the app to the women when they are sent to a DXA scan at the hospital. When aiming to transform healthcare by designing and developing an mHealth app, it is crucial that there is a willingness to implement the solution. When we invited relevant stakeholders to participate in the team, everyone agreed to participate. Even then, the support of transforming the healthcare services within the field of early stage osteoporosis was high. However, the selection of participants in the team has some limitations, since not every user can be involved in a participatory design study. It is simply not practical or manageable to involve every user in workshops and laboratory tests and feedback on prototypes. We cannot be sure that we have selected the best representatives, though we have experienced a great support and interest in the mHealth app at the department at the University Hospital. This shows us that we have hit the nail on the head in some way.

Clemensen at al. recommend among others when conducting a participatory design study that the researcher leading the project should be open-minded about various suggestions that arise [23]. We have tried our best to be open-minded. However, the project has both time and financial resource limitations. Therefore, we could not accommodate every need expressed by the users.

## 5. Conclusions

Results from the workshops and laboratory tests demonstrated that participatory design is a viable approach when developing an mHealth app for women diagnosed with osteoporosis without preceding fractures to support them in treatment decision-making and self-management of the disease. Especially, the use of feedback from the users in the iterative process in the development phase is of great importance. Additionally, the opportunity to engage users and designers in workshops and laboratory tests to enable mutual learning is of great importance. Furthermore, we found that regular member-checks like interviews of users helped us to identify challenges associated with providing healthcare services through an app.

“My Osteoporosis Journey” is the first app developed for women who are diagnosed with osteoporosis without preceding fractures based on their identified needs. The app is to be used in addition to regular osteoporosis care provided by the hospital and in general practice. It is a new way of providing healthcare, and we hope that the app is experienced as a useful support for the women. To test “My Osteoporosis Journey” (phase 3) in a real-life setting, a usability test will be conducted. Thirty women will be invited to participate, and their experiences and feedback when using the app will be collected through semi-structured interviews. This will be the topic for future papers.

## Figures and Tables

**Table 1:** Participants in the participatory design team, their characteristics, and contributions in the workshops and laboratory tests.

Participants and Their Roles	Participants Characteristics	Workshop Attendance	Lab. Test Attendance
Women newly diagnosed with osteoporosis (*n* = 6). Generate ideas and express needs from a user perspective	Age 56, referred to DXA scan due to known familial predisposition	1.3	
	Age 65, asked for a DXA scan due to known familial predisposition	1.3	
	Age 54, referred to DXA scan due to known familial predisposition	1	
	Age 58, asked for a DXA scan due to known familial predisposition	1.3	
	Age 56, asked for a DXA scan due to known familial predisposition	1.3	
	Age 52, referred to DXA scan due to known familial predisposition	1.3	
Healthcare professionals (*n* = 8) Generate ideas and express needs from a healthcare professional perspective	Female, age 48, hospital physician, Dep. of Endocrinology	1.2	
	Female, age 57, leading hospital physician, dep. of endocrinology	1.2	1.2
	Male, age 40, GP	1	
	Male, age 54, GP, Professor in health science	2	1
	Female, age 41, nurse, specialized within osteoporosis	1.2	1
	Female, age 42, laboratory specialist, dep. of endocrinology	1	2
	Female, age 41, laboratory specialist, dep. of endocrinology	1.2	1.2
	Female, age 57, dietician, The Danish Osteoporosis Foundation	1	
Researchers (*n* = 2) Facilitate the PD ^1^ process	Female, age 53, Professor in health science, experienced in PD ^1^	1.2.3	2
	Female, age 39, project leader, PhD student within health research	1.2.3	1.2
App designers (*n* = 2) Design and development of the mHealth app	Male 39, experienced in mHealth development		2
	Male 38, experienced in mHealth development	1.2	1.2

^1^ PD = participatory design.

## References

[1] Hood M., Wilson R., Corsica J., Bradley L., Chirinos D., Vivo A. (2016). What do we know about mobile applications for diabetes self-management? A review of reviews. J. Behav. Med..

[2] Becker S., Miron-Shatz T., Schumacher N., Krocza J., Diamantidis C., Albrecht U.V. (2014). mHealth 2.0: Experiences, Possibilities, and Perspectives. JMIR mHealth uHealth.

[3] Whitehead L., Seaton P. (2016). The Effectiveness of Self-Management Mobile Phone and Tablet Apps in Long-term Condition Management: A Systematic Review. J. Med. Internet Res..

[4] Kanis J.A., Svedbom A., Harvey N., McCloskey E.V. (2014). The osteoporosis treatment gap. J. Bone Miner. Res..

[5] Kanis J.A., McCloskey E.V., Johansson H., Cooper C., Rizzoli R., Reginster J.Y. (2013). European guidance for the diagnosis and management of osteoporosis in postmenopausal women. Osteoporos. Int..

[6] Sozen T., Ozisik L., Basaran N.C. (2017). An overview and management of osteoporosis. Eur. J. Rheumatol..

[7] Vestergaard P., Rejnmark L., Mosekilde L. (2005). Osteoporosis is markedly underdiagnosed: A nationwide study from Denmark. Osteoporos. Int..

[8] Leslie W.D., Tsang J.F., Caetano P.A., Lix L.M., Manitoba Bone Density Program (2007). Effectiveness of bone density measurement for predicting osteoporotic fractures in clinical practice. J. Clin. Endocrinol. Metab..

[9] Cooper C., Reginster J.Y., Cortet B., Diaz-Curiel M., Lorenc R.S., Kanis J.A., Rizzoli R. (2012). Long-term treatment of osteoporosis in postmenopausal women: A review from the European Society for Clinical and Economic Aspects of Osteoporosis and Osteoarthritis (ESCEO) and the International Osteoporosis Foundation (IOF). Curr. Med. Res. Opin..

[10] Besser S.J., Anderson J.E., Weinman J. (2012). How do osteoporosis patients perceive their illness and treatment? Implications for clinical practice. Arch. Osteoporos..

[11] Nielsen D., Huniche L., Brixen K., Sahota O., Masud T. (2013). Handling knowledge on osteoporosis—A qualitative study. Scand. J. Caring Sci..

[12] Weston J.M., Norris E.V., Clark E.M. (2011). The invisible disease: Making sense of an osteoporosis diagnosis in older age. Qual. Health Res..

[13] Hansen C., Konradsen H., Abrahamsen B., Pedersen B.D. (2014). Women’s experiences of their osteoporosis diagnosis at the time of diagnosis and 6 months later: A phenomenological hermeneutic study. Int. J. Qual. Stud. Health Well-Being.

[14] Hansen C.A., Abrahamsen B., Konradsen H., Pedersen B.D. (2017). Women’s lived experiences of learning to live with osteoporosis: A longitudinal qualitative study. BMC Women's Health.

[15] Hiligsmann M., Kanis J.A., Compston J., Cooper C., Flamion B., Bergmann P., Body J., Boonen S., Bruyere O., Devogelaer J. (2013). Health technology assessment in osteoporosis. Calcif. Tissue Int..

[16] Huybrechts K.F., Ishak K.J., Caro J.J. (2006). Assessment of compliance with osteoporosis treatment and its consequences in a managed care population. Bone.

[17] Free C., Phillips G., Galli L., Watson L., Felix L., Edwards P., Patel V., Haines A. (2013). The effectiveness of mobile-health technology-based health behaviour change or disease management interventions for health care consumers: A systematic review. PLoS Med..

[18] Krishna S., Boren S.A., Balas E.A. (2009). Healthcare via cell phones: A systematic review. Telemed. eHealth.

[19] Peeters J.M., Wiegers T.A., Friele R.D. (2013). How technology in care at home affects patient self-care and self-management: A scoping review. Int. J. Environ. Res. Public Health.

[20] Slomian J., Appelboom G., Ethgen O., Reginster J.Y., Bruyere O. (2014). Can new information and communication technologies help in the management of osteoporosis?. Women's Health.

[21] Kushniruk A., Nohr C. (2016). Participatory Design, User Involvement and Health IT Evaluation. Stud. Health Technol. Inform..

[22] Garne Holm K., Brodsgaard A., Zachariassen G., Smith A.C., Clemensen J. (2017). Participatory design methods for the development of a clinical telehealth service for neonatal homecare. SAGE Open Med..

[23] Clemensen J., Larsen S.B., Kyng M., Kirkevold M. (2007). Participatory design in health sciences: Using cooperative experimental methods in developing health services and computer technology. Qual. Health Res..

[24] Noergaard B., Sandvei M., Rottmann N., Johannessen H., Wiil U., Schmidt T., Pedersen S.S. (2017). Development of a Web-Based Health Care Intervention for Patients With Heart Disease: Lessons Learned From a Participatory Design Study. JMIR Res. Protoc..

[25] Cnossen I.C., van Uden-Kraan C.F., Eerenstein S.E., Rinkel R.N., Aalders I.J., van den Berg K., de Goede C.J., van Stijgeren A.J., Cruijff-Bijl Y., de Bree R. (2015). A Participatory Design Approach to Develop a Web-Based Self-Care Program Supporting Early Rehabilitation among Patients after Total Laryngectomy. Folia Phoniatr. Logop..

[26] Laidlaw R., Dixon D., Morse T., Beattie T.K., Kumwenda S., Mpemberera G. (2017). Using participatory methods to design an mHealth intervention for a low income country, a case study in Chikwawa, Malawi. BMC Med. Inform. Decis. Mak..

[27] Clemensen J., Rothmann M.J., Smith A.C., Caffery L.J., Danbjorg D.B. (2016). Participatory design methods in telemedicine research. J. Telemed. Telecare.

[28] Clemensen J. (2006). Pervasive Healthcare: Home Treatment of Patients with Diabetic Foot Ulcers.

[29] Andersen T.O., Bansler J.P., Kensing F., Moll J. (2017). From Prototype to Product: Making Participatory Design of mHealth Commercially Viable. Stud. Health Technol. Inform..

[30] Grönvall E., Kyng M. (2012). On participatory design of home-based healthcare. Cogn. Technol. Work..

[31] Simonsen J., Robertson T. (2013). Routledge International Handbook of Participatory Design.

[32] Clemensen J., Larsen S.B., Kirkevold M., Ejskjaer N. (2008). Treatment of diabetic foot ulcers in the home: Video consultations as an alternative to outpatient hospital care. Int. J. Telemed. Appl..

[33] Jakobsen P.R., Hermann A.P., Soendergaard J., Wiil U.K., Dixon R.F., Clemensen J. (2018). Left in Limbo-experiences and needs among postmenopausal women newly diagnosed with osteoporosis without preceding osteoporotic fractures: A qualitative study. Post Reprod. Health.

[34] Gray D., Brown S., Macanufo J. (2010). Game Storming. A Playbook for Innovators, Rulebreakers, and Changemakers.

[35] Neuhauser L. (2017). Integrating Participatory Design and Health Literacy to Improve Research and Interventions. Stud. Health Technol. Inform..

[36] Spinuzzi C. (2005). The Methodology of Participatory Design. Tech. Commun..

[37] Boe Danbjorg D., Wagner L., Kristensen B.R., Clemensen J. (2015). Nurses’ experience of using an application to support new parents after early discharge: An intervention study. Int. J. Telemed. Appl..

[38] Clemensen J., Larsen S.B., Kirkevold M., Ejskjaer N. (2007). Telemedical teamwork between home and hospital: A synergetic triangle emerges. Stud. Health Technol. Inform..

[39] Kanis J.A., Johnell O., De Laet C., Johansson H., Oden A., Delmas P., Eisman J., Fujiwara S., Garnero P., Kroger H. (2004). A meta-analysis of previous fracture and subsequent fracture risk. Bone.

